# The FlbA-regulated predicted transcription factor Fum21 of *Aspergillus niger* is involved in fumonisin production

**DOI:** 10.1007/s10482-017-0952-1

**Published:** 2017-09-30

**Authors:** David Aerts, Esther E. Hauer, Robin A. Ohm, Mark Arentshorst, Wieke R. Teertstra, Christopher Phippen, Arthur F. J. Ram, Jens C. Frisvad, Han A. B. Wösten

**Affiliations:** 1 Microbiology, Department of Biology, Utrecht University, Padualaan 8, 3584 CH Utrecht, The Netherlands; 2 Department of Molecular Microbiology and Biotechnology, Institute of Biology Leiden, Leiden University, Sylviusweg 72, 2333 BE Leiden, The Netherlands; 3 Department of Systems Biology, Technical University of Denmark, 2800 Lyngby, Denmark

**Keywords:** Asexual development, Aspergillus, Fumonisin, Fungus, Mycotoxin, Secondary metabolism, Protein secretion

## Abstract

*Aspergillus niger* secretes proteins throughout the colony except for the zone that forms asexual spores called conidia. Inactivation of *flbA* that encodes a regulator of G-protein signaling results in colonies that are unable to reproduce asexually and that secrete proteins throughout the mycelium. In addition, the Δ*flbA* strain shows cell lysis and has thinner cell walls. Expression analysis showed that 38 predicted transcription factor genes are differentially expressed in strain Δ*flbA*. Here, the most down-regulated predicted transcription factor gene, called *fum21*, was inactivated. Growth, conidiation, and protein secretion were not affected in strain Δ*fum21*. Whole genome expression analysis revealed that 63 and 11 genes were down- and up-regulated in Δ*fum21*, respectively, when compared to the wild-type strain. Notably, 24 genes predicted to be involved in secondary metabolism were down-regulated in Δ*fum21*, including 10 out of 12 genes of the fumonisin cluster. This was accompanied by absence of fumonisin production in the deletion strain and a 25% reduction in production of pyranonigrin A. Together, these results link FlbA-mediated sporulation-inhibited secretion with mycotoxin production.

**Electronic supplementary material:**

The online version of this article (doi:10.1007/s10482-017-0952-1) contains supplementary material, which is available to authorized users.

## Introduction

The genus *Aspergillus* consists of more than 300 species (Samson et al. 2014) that are among the most abundant fungi on the globe. Aspergilli degrade plant waste and as such play a role in carbon cycling in nature. Moreover, the genus includes opportunistic pathogens of plants, animals, and humans (Krijgsheld et al. 2013a). Enzymes secreted by *Aspergillus* play an important role in degradation of organic material and pathogenicity. The property to secrete high levels and a diversity of enzymes make aspergilli such as *Aspergillus niger* important cell factories for the production of proteins and metabolites (Meyer et al. 2011; Wösten et al. 2013).

Aspergilli form a mycelium consisting of a network of hyphae that grow at their apex and that branch subapically. The mycelium grows initially vegetative but at a certain moment asexual development is initiated (Krijgsheld et al. 2013a). The resulting conidia are the only spore type that are produced by *A. niger.* Conidiation starts with the formation of thick aerial hyphae called stalks. When a stalk has reached a certain height, its tip swells to form a vesicle. In many aspergilli, this structure buds resulting in a layer of metulae. The metulae in turn form a layer of phialides, from which chains of conidia develop. These spores are heterogeneous in composition, water dispersal efficiency, and germination rate (Teertstra et al. 2017) and give rise to new mycelia.

The zone forming the asexual conidia within an *A. niger* colony does not secrete proteins, while non-sporulating zones do release proteins into their environment (Krijgsheld et al. 2013b). Preventing conidiation of the colony by covering it with a polycarbonate membrane does not impact the spatial secretion pattern indicating that the capacity to sporulate but not the conidiation process itself inhibits secretion. FlbA was shown to impact spatial secretion in the colony (Krijgsheld et al. 2013b). This protein regulates the Gα subunit FadA by activating its GTPase activity. By doing so, it promotes asexual development and inhibits vegetative growth and autolysis (Wieser et al. 1994; Yu et al. 1996). Conidiation is abolished in the Δ*flbA* strain of *A. niger* and, as a consequence, protein secretion takes place throughout the colony (Krijgsheld et al. 2013b). Moreover, the cell wall of Δ*flbA* is thinner and intracellular proteins can be found in the culture medium, indicative of cell lysis. The Δ*flbA* strain therefore has a pleiotropic phenotype. Inactivation of *flbA* is accompanied by differential expression of 38 transcription factor genes, of which 18 are down-regulated and 20 are up-regulated (Krijgsheld and Wösten 2013). These downstream regulatory genes may impact one or more of the processes affected by FlbA. Here, the role of the most down-regulated predicted transcription factor gene, *fum21,* was studied. It is shown that *fum21* regulates production of the mycotoxins fumonisins. Thus, sporulation-inhibited protein secretion is linked to production of secondary metabolites via FlbA.

## Materials and methods

### Strains and culture conditions

*A. niger* strain MA234.1 (transient ku70::amdS) (Park et al. 2016) and its derivatives were grown at 30 °C using minimal medium (MM) (de Vries et al. 2004) containing 25 mM xylose as a carbon source and either (MM-XA) or not (MM-X) containing 1.5% agar. Alternatively, strains were grown on Czapek Yeast Auto-lysate (CYA) agar (Frisvad and Samson 2004), Yeast Extract Sucrose (YES) agar (Frisvad and Samson 2004), or transformation medium (TM; MM containing 0.5% yeast extract, 0.2% casaminoacids, and 25 mM glucose, pH 6)(Kusters-van Someren et al. 1991).

Conidia were harvested from 3-day-old MM-XA cultures. Liquid cultures inoculated with 5 × 10^8^ spores were pre-grown for 16 h at 200 rpm in 300 ml TM in 500 ml Erlenmeyer flasks. After 16 h, 10 g wet weight mycelium was harvested, washed with 0.9% NaCl, and transferred to a 1 l Erlenmeyer flask containing 150 ml MM-X. The culture was shaken at 250 rpm and 30 °C for 4 h (RNA sequencing) or 24 h (SDS-PAGE).

For growth on agar media, strains were inoculated directly on the medium or grown in a layer of 1.25% agarose in between two perforated polycarbonate membranes (pores of 0.1 µm, diameter 76 mm; Profiltra, Almere, The Netherlands) (Wösten et al. 1991). These sandwiched cultures were inoculated in the center of the agarose layer by placing a 2 µl drop containing 10^4^ conidia. The upper polycarbonate membrane was placed on top of the agarose layer 24 h after inoculation to prevent spreading of conidia.

### Inactivation and complementation constructs of *fum21*

For the construction of the *fum21* deletion strain, 5′ and 3′ flanks of the gene were amplified from genomic DNA by PCR using primer pairs 1/2 and 3/4, respectively (Supplemental Table 1). The hygromycin resistance gene *hph* was amplified from vector pAN7.1 (Punt et al. 1987) using primer pair 5/6 (Supplemental Table 1). Split marker fragments of this selection marker were created by fusion PCR (Arenthorst et al. 2015) using primer pairs 1/7 and 4/8 (Supplemental Table 1) for the 5′ and 3′ fragment, respectively.

For the construction of the *fum21* complementation construct, the hygromycin resistance cassette contained in pAN7.1 was amplified using primers 9 and 10 (Supplemental Table 1) that contain XbaI sites at their 5′ ends. The amplified 3 kb fragment that had a shorter promoter and terminator region as compared to pAN7.1 was cloned in the XbaI site of pUC20, resulting in vector pWR11. Gene *fum21* with 1048 bp promoter and 444 bp terminator sequence was amplified from MA234.1 genomic DNA using Phusion polymerase (Thermo Fisher Scientific, Waltham, MA, USA) and primer pair 11/12 (Supplemental Table 1). The amplified fragment was inserted in the SmaI site of pWR11 by using the InFusion^®^ HD Cloning Kit (Clontech, Mountain View, CA, USA). This resulted in vector pDA1 containing the hygromycin resistance cassette and gene *fum21*.

### Transformation of *A. niger*

Transformation of *A. niger* was performed according to de Bekker et al. (2009). For inactivation of *fum21*, the split marker fragments were transformed to strain MA234.1. Transformants were purified twice on MM-XA containing hygromycin. Deletion of *fum21* was verified by Southern blot analysis using HindIII digested genomic DNA (Supplemental Fig. 1). For complementation, the disrupted *kusA* gene in the Δ*fum21* strain was first restored by selecting AmdS loop-out strains on 5′ fluoroacetamide (Carvalho et al. 2010) to facilitate ectopic integration. Since the *fum21* deletion strain was already hygromycin resistant, the resulting Δ*fum21 kusA* ^+^ strain was complemented by co-transforming vectors pDA1 and pMA357. The latter pJet1.2 (Thermo Fisher Scientific) based vector contains the *amdS* gene and 3′ regulatory sequences of *Aspergillus nidulans* under control of the *gpdA* promoter. The amdS expression cassette (Meyer et al. 2007) was PCR amplified with primer pair 13/14 (Supplemental Table 1). Selection was done on MM containing 0.95 M sucrose, 15 mM CsCl, and 10 mM acetamide as sole nitrogen source. Integration of the wild-type copy of the gene was confirmed by Southern blot analysis (Supplemental Fig. 1).

### RNA sequencing and analysis

Mycelium of biological duplicates of liquid cultures pre-grown in TM and transferred to MM-X was frozen in liquid nitrogen and ground for 1 min at 25 Hz with a Tissue Lyzer II (Qiagen, Venlo, The Netherlands). Samples were taken up in 1 ml TRIzol reagent (Invitrogen, Bleiswijk, The Netherlands) and incubated for 5 min at room temperature (RT). 0.2 ml chloroform was added and samples were centrifuged for 15 min at 4 °C and 12,000×*g* after 2 min incubation at RT. Total RNA was precipitated from the resulting aqueous phase by addition of 0.5 ml isopropanol, incubation at RT for 10 min, and centrifugation for 10 min at 4 °C and 12,000×*g*. RNA was washed with 1 ml 75% ethanol, left to dry, and resuspended in RNAse-free water. RNA was purified using the NucleoSpin^®^ RNA kit (Macherey-Nagel, Düren, Germany). Concentration and purity of RNA was checked using the Nanodrop ND-1000 (Thermo Fisher Scientific).

Library preparation, cluster generation, and sequencing of cDNA were performed by ServiceXS (Leiden, The Netherlands) using Illumina sequencing. The reads are deposited in NCBI GEO with accession number GSE93990. Tophat version 2.1.13 (Trapnell et al. 2009) was used to align sequence reads to the Aspni7 version of the *A. niger* ATCC 1015 genome (Andersen et al. 2011), which was obtained from Mycocosm (Grigoriev et al. 2014). Functional annotation of the predicted genes was described previously (Teertstra et al. 2017). Cuffdiff (version 2.2.1), which is part of Cufflinks (Trapnell et al. 2010), was used to identify reads mapping to predicted genes and to identify differentially expressed genes. The bias correction method was used while running Cuffdiff (Roberts et al. 2011). The expression level of each predicted gene was normalized to fragments per kilobase of exon model per million fragments (FPKM). In addition to Cuffdiff’s requirements for differential expression the following requirements were applied: a  ≥ 2-fold change and a minimal expression level of 4 FPKM in at least one of the samples. The quality of these results was analyzed using CummeRbund (Goff et al. 2013).

### Q-PCR

Expression of *fum10* (proteinID 1117227), *fum8* (ProteinID 1117230), and *fum1* (ProteinID 1162446) in *A. niger* wild type, Δ*fum21,* and 4 complemented strains was assessed by Q-PCR using beta-tubulin (ProteinID 208263) and 18S rRNA (GenBank sequence ID KC545869.1) as reference genes. Total RNA was isolated as described above from biological duplicates of CYA- and YES-grown sandwiched colonies, after which it was purified using NucleoSpin^®^ RNA (Macherey-Nagel, Düren, Germany) and reverse transcribed using the QuantiTect Reverse Transcription Kit (Qiagen, Venlo, The Netherlands). The cDNA (1 ng) was used for SYBR Green Q-PCR using 200 nM of the primer pairs 15/16 for *fum10*, (efficiency 98.7%), 17/18 for *fum8* (efficiency 104.4%), 19/20 for *fum1* (efficiency 109.7%), 21/22 for beta-tubulin (efficiency 102.1%), and 23/24 for 18S (efficiency 95.8%) (Supplemental Table 1). No-template controls (NTCs) were included as a negative control and total reaction volumes were 10 µl. Samples were run on a ViiA™ 7 Real-Time PCR System (Applied Biosystems, Wilmington DE, USA) and analyzed using the ΔΔCt method.

### SDS-PAGE

Proteins in culture medium were precipitated overnight at − 20 °C after adding 4 volumes of acetone. They were pelleted twice for 30 min at 4 °C and 21,000×*g* with an intermediate washing step using − 20 °C acetone. After drying the pellets, sample buffer (125 mM Tris pH 6.8, 4% sodium dodecyl sulfate (SDS), 17.4% glycerol, 5% β-mercaptoethanol, 200 µg/ml bromophenol blue) was added resulting in a 20-fold volume reduction when compared to the culture medium. Samples were incubated at 100 °C for 10 min and proteins were separated in 12.5% SDS-PAGE gels using Pierce™ Prestained Protein Molecular Weight Marker (Thermo Fisher Scientific) as reference. Gels were fixed with 50% methanol and 10% acetic acid for 10 min, stained overnight with 0.1% Coomassie Brilliant Blue R-250, and destained with 25% methanol, 10% acetic acid. Gels were imaged using the Universal Hood III with Image Lab software (Bio-Rad Laboratories BV, Veenendaal, The Netherlands).

### Localization of protein secretion

Protein secretion was monitored as described (Krijgsheld et al. 2013b) by labelling sandwiched colonies that had been grown on MM-XA for 6 days and transferred for 24 h to fresh MM-XA containing ^14^C-amino acids (NEC-850E amino acid mixture, L-[^14^C(U)]-, Perkin Elmer, Waltham MA, USA).

### Protease activity

*A. niger* was grown as a sandwiched colony (see above) on MM containing 33% skimmed milk and 1.5% agar. After 5 days of growth, the sandwiched culture was removed and presence of halos monitored.

### Secondary metabolite profiling

CYA and YES plates were inoculated in duplicate with 10^4^ conidia. After 7 days of growth at 25 °C, 56-mm wide mycelial plugs were taken along the diameter of the colony and freeze-dried. Secondary metabolites were extracted in duplicate from the plugs with ethylacetate/dichloromethane/methanol (3:2:1, vol/vol/vol) with 1% formic acid using ultrasonic treatment for 50 min (Smedsgaard 1997). The extracts were transferred to a 2 ml dram vial and taken up in 300 µl methanol after removing the organic solvents by evaporation. Pyranonigrin A, pyranonigrin X (i.e. pyranonigrin B, C, D, E, or S), kotanin, BMS 192548, aurasperone B, tensidol B, and funalenone were analyzed using ultra-high performance liquid chromatography (UHPLC) (Houbraken et al. 2012). These compounds were identified using diode array detection (UV spectra from 190-600 nm) with purified compounds as standard. The relative quantity of the metabolites was estimated based on absorption at 210 nm (milli absorption units, mAU). Fumonisin B_2_, B_4_, and B_6_ were quantified by UHPLC-High Resolution Mass Spectrometry (UHPLC-HRMS) using an Agilent Infinity 1290 UHPLC system (Agilent Technologies, Santa Clara, CA, USA) equipped with a diode array detector. Separation was obtained on an Agilent Poroshell 120 phenyl-hexyl column (2.1 × 250 mm, 2.7 μm) with a linear gradient consisting of water (A) and acetonitrile (B) both buffered with 20 mM formic acid, starting at 10% B, increased to 100% B in 15 min, and held for 2 min at this composition, returned to 10% B in 0.1 min and held for 3 min at this composition (0.35 ml min^−1^, 60 °C). An injection volume of 1 μl was used. MS detection was performed on an Agilent 6540 QTOF MS equipped with Agilent Dual Jet Stream electrospray ion source with a drying gas temperature of 250 °C, gas flow of 8 l min^−1^, sheath gas temperature of 300 °C and flow of 12 l min^−1^. Capillary and nozzle voltage were set at 4000 and 500 V, respectively. Mass spectra were recorded at 10, 20, and 40 eV as centroid data for m/z 85–1700 in MS mode and m/z 30–1700 in MS/MS mode, with an acquisition rate of 10 spectra/s. Lock mass solution in 70:30 methanol: water was infused in the second sprayer using an extra LC pump at a flow of 15 μl min^−1^ using a 1:100 splitter. The solution contained 1 μM tributylamine (Sigma-Aldrich) and 10 μM Hexakis (2,2,3,3-tetrafluoropropoxy)phosphazene (Apollo Scientific Ltd., Cheshire, UK) as lock masses. The [M + H] + ions (m/z 186.2216 and 922.0098, respectively) of both compounds was used (Kildgaard et al. 2014; Klitgaard et al. 2014).

## Results

### Functional characterization of* fum21*

Thirty eight predicted transcription factor genes are differentially expressed in xylose-grown colonies of Δ*flbA* when compared to wild type (Krijgsheld and Wösten 2013). Of these genes, An01g06900 (Cerqueira et al. 2014) is the most down-regulated predicted transcription factor gene with a 22, 42, and 31 fold-change in the central, middle, and the outermost concentric zone of the colony, respectively (Krijgsheld and Wösten 2013). This gene showed a bi-directional hit with the fumonisin regulator *fum21* of *Fusarium* (Proctor et al. 2013) showing 29% identity at amino acid level and sharing the GAL4 DNA binding domain and the Middle Homology Region (MHR) domain (Supplemental Fig. 2). The fact that An01g06900 (i.e. *fum21*) is located in the predicted fumonisin gene cluster of *A. niger* (Supplemental Fig. 2) supports a role of this gene in fumonisin production.

**Fig. 1:**
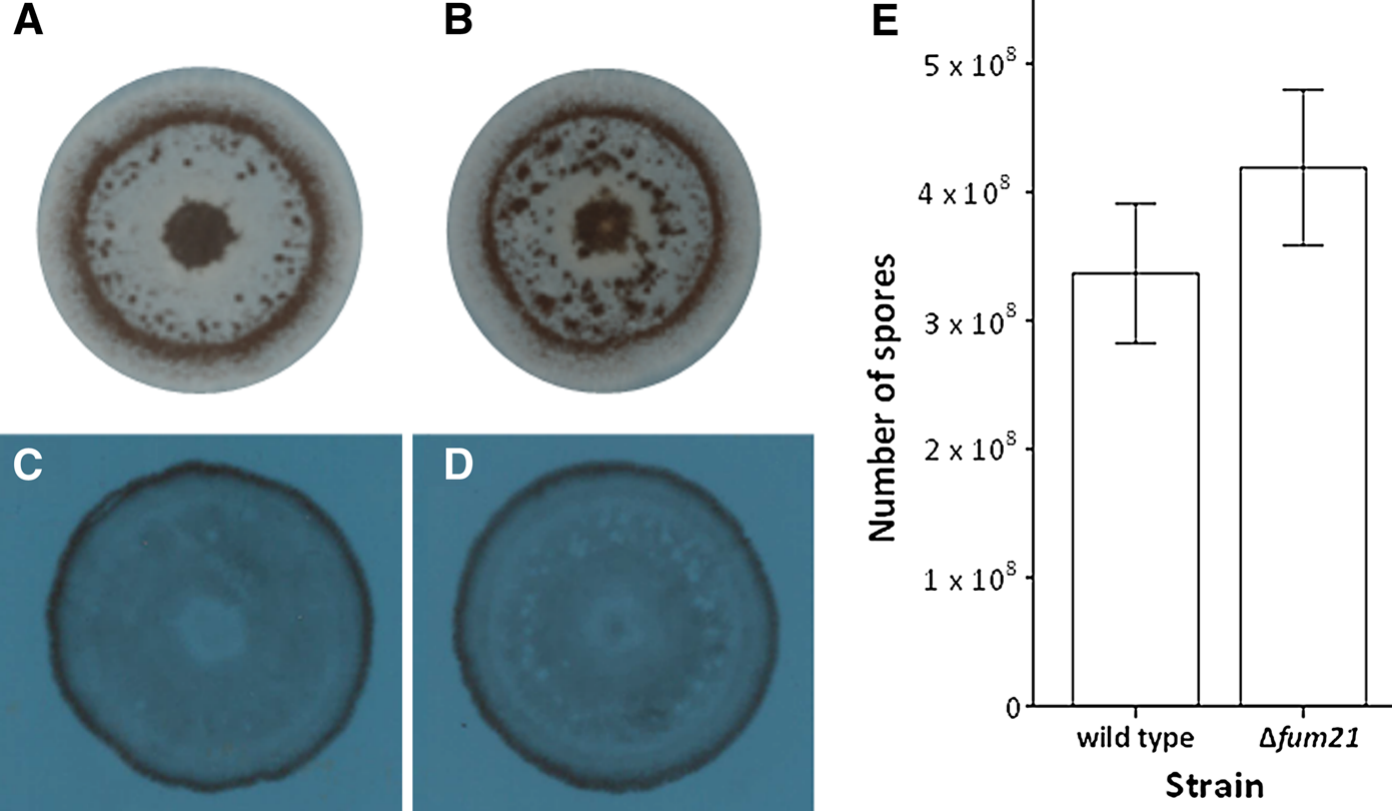
Spatial distribution of sporulation (**a**, **b**), protein secretion (**c**, **d**), and the number of spores that were produced (**e**) in 8-day-old xylose-grown colonies of the wild-type strain MA234.1 (**a**, **c**) and Δ*fum21* (**b**, **d**). Secretion was monitored by immobilizing ^14^C-labeled secreted proteins on a PVDF membrane that had been placed underneath the colony. Localization and quantification of sporulation was monitored 48 h after removal of the upper membrane of sandwiched colonies

Gene *fum21* was deleted in *A. niger* strain MA234.1, resulting in strain Δ*fum21* (Supplemental Fig. 1). Growth and conidiation were not affected in the deletion strain. Biomass of 7-day-old wild-type and Δ*fum21* sandwiched colonies was 17.20 ± 3.4 mg and 17.43 ± 2.0 mg (± SD, *n* = 7, *p* > 0.05), while colony diameter was 4.6 ± 0.63 cm and 4.9 ± 0.29 cm (± SD, *n* = 6, *p* > 0.05). Conidiation in the two strains took place in the sub-peripheral zone and the center of the colonies (Fig. 1a, b). These sporulation zones did not secrete proteins (Fig. 1c, d). SDS-PAGE protein profiles obtained in liquid cultures were not different between the strains (data not shown). Degradation of skimmed milk (data not shown) and the number of spores that were produced also did not differ (Fig. 1e). These data show that conidiation and sporulation-inhibited secretion are still functional in Δ*fum21*.

**Fig. 2:**
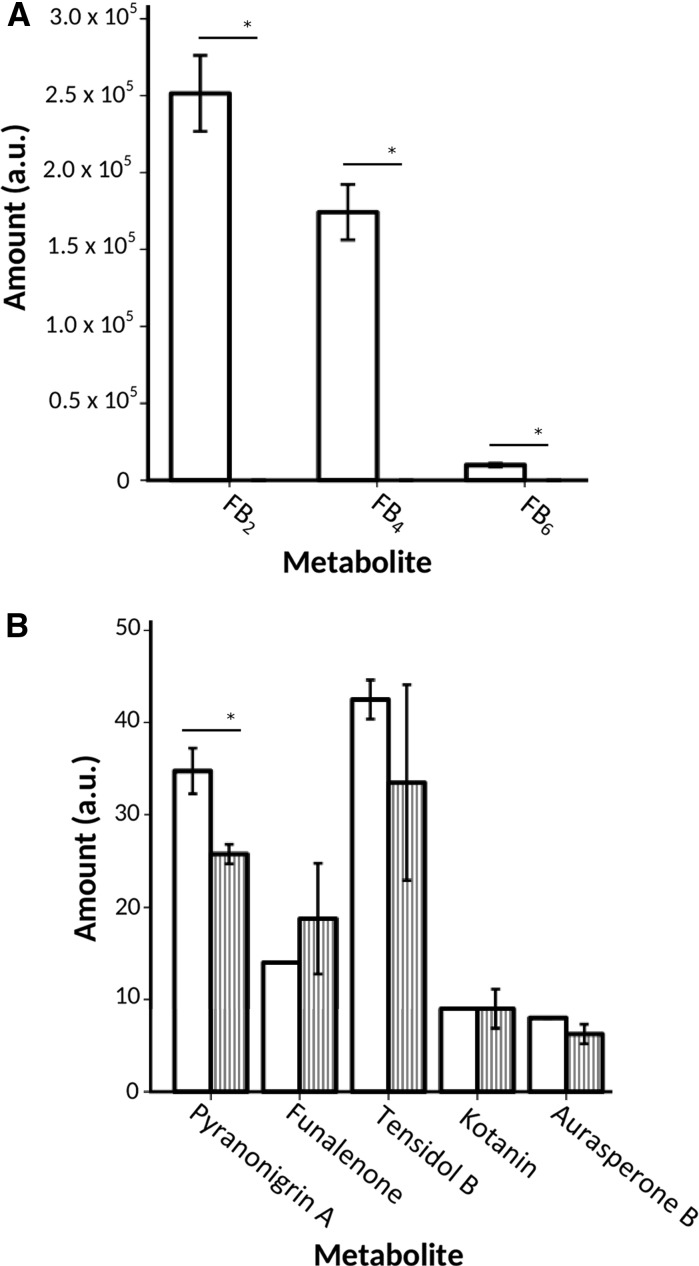
Amount of fumonisin B_2_, B_4_, and B_6_ (FB_2_, FB_4_, and FB_6_, respectively) (**a**) and other secondary metabolites (**b**) in arbitrary units (a.u.), produced by the wild-type strain MA234.1 (open bars) and Δ*fum21* (gray shaded bars) in CYA medium. Asterisk indicates significant differences (p ≤ 0.05) between the two strains indicated by the horizontal line below the asterisk

Strain Δ*fum21* and its progenitor were grown for 7 days on CYA. Fumonisin B_2_, B_4_, and B_6_ could be extracted from mycelial plugs from different zones of wild-type colonies. In contrast, Δ*fum21* did not produce these metabolites (Fig. 2a). Similar results were obtained when colonies were grown on YES medium (data not shown). Production of pyranonigrin A was also reduced by 25% in the deletion strain but production of other secondary metabolites was not affected when Δ*fum21* and the wild type were grown on CYA (Fig. 2b) and YES (data not shown) medium.

### Gene expression analysis

Gene expression in Δ*fum21* and the wild-type progenitor strain was assessed by RNA sequence analysis after growth in TM for 16 h followed by transfer to MM-X for 4 h. This analysis revealed that only 74 genes were differentially expressed in Δ*fum21* relative to the wild type. Of these genes, 63 were down-regulated and 11 were up-regulated in the deletion strain (Table 1; Supplemental Table 2). Of the up-regulated genes, 7 have a predicted signal sequence for secretion including genes encoding lipases, a protease, a cellobiohydrolase and an anti-fungal protein. Of the down-regulated genes, 12 genes encode a protein without annotation and 24 genes are predicted to be involved in secondary metabolism. Of the latter genes, 20 are located in the secondary metabolism clusters 1 (3 out of 21 genes down-regulated), 12 (4 out of 13 genes down-regulated), 70 (3 out of 6 genes down-regulated), and 15 (10 out of 12 genes down-regulated). The secondary metabolites produced by the proteins encoded by clusters 1 and 12 are unknown but clusters 70 and 15 are predicted to be involved in TAN-1612 and fumonisin production, respectively (Li et al. 2011; Khaldi and Wolfe 2011). The down-regulated genes in the fumonisin cluster included *fum10*, *fum8,* and *fum1* (Table 2), which are predicted to be involved in the early catalytic steps of fumonisin synthesis in *Fusarium* (Proctor et al. 2003). Q-PCR analysis showed that their expression was absent in Δ*fum21* of *A. niger* and restored in two complemented strains (data not shown).

**Table 1:** Differentially expressed genes in liquid shaken cultures of Δ*fum21* and the wild-type strain (for full dataset, see Supplemental Table 2)

Protein id	Δ*fum21*	Wild type	Functional annotation
Down-regulated genes in Δfum21			
1166045	0	13.21	AAA+ -type ATPase
1182124	0	4.89	Major facilitator superfamily transporter
225717	0	29.92	*fum21*
1117227	0.30	519.60	Peroxisomal acyl-CoA synthetase; fum10 orthologue
1117230	0.25	368.97	α-Oxoamine synthase; serine palmitoyltransferase; fum8 orthologue
1182116	0.39	573.20	Fe-containing alcohol dehydrogenase type IV; fum7 orthologue
1142053	1.09	1433.24	No annotation
1162446	0.25	167.04	Polyketide synthase; fum1 orthologue
1162442	2.19	913.32	NAD-dependent epimerase/dehydratase; fum13 orthologue
1101614	0.98	236.55	Cytochrome p450; fum6 orthologue
1142051	8.34	1607.79	No annotation
1166044	2.75	347.93	No annotation
1172265	1.62	191.62	Oxidoreductase
1162443	6.66	564.86	CoA-dependent acyltransferase; fum14 orthologue
1186369	0.57	19.06	Ca^2+^-modulated nonselective cation channel polycystin
51907	3.73	110.86	Predicted 3-ketosphinganine reductase
1181633	0.49	12.69	SWI-SNF chromatin-remodeling complex protein
1082505	1.48	32.99	Major facilitator superfamily transporter
1142861	1.59	33.92	Chloroperoxidase
1159889	0.93	17.88	O-methyltransferase
1087288	2.37	39.12	Taurine catabolism dioxygenase TauD
45784	1.70	25.17	Ca^2+^-modulated nonselective cation channel polycystin
1169210	5.86	72.09	Glutathione S-transferase-like protein
1116476	2.31	28.18	CDR ABC transporter
1089440	2.52	29.10	Major facillitator superfamily transporter
1112167	0.60	6.38	Polyketide synthase AdaA
1181632	2.79	26.80	No annotation
1115620	1.27	12.02	C-type lectin
189113	8.60	80.47	NmrA-like family protein
1005100	2.33	21.65	No annotation
1186279	20.64	191.85	No annotation
1103854	1.34	11.77	Glycosyl transferase
1139199	4.02	34.97	Mono-oxygenase, FAD-binding/aromatic ring hydroxylase
1186352	2.74	23.65	Molecular chaperone
1109756	1.59	13.54	Kinesin-related protein
1187549	1.99	16.75	Integral membrane protein
1125454	0.71	5.80	Dihydroxy-acid dehydratase
1184525	3.92	31.30	Non-ribosomal peptide synthetase
1124090	2.40	18.02	Tryptophan synthase
1015414	1.95	14.50	Short chain dehygrogenase
1139200	33.67	239.67	AdaD
1187587	2.19	15.55	AAA+ -type ATPase
1152279	4.10	28.71	Major facilitator superfamily transporter
1162650	21.21	146.94	Aegerolysin
1200239	5.00	34.01	NmrA-like family protein
1223842	1.36	9.10	hypothetical FAD/FMN-containing dehydrogenase
1186845	0.79	5.13	No annotation
1187028	192.14	1228.87	No annotation
1181350	3.78	21.89	*Aspergillus kawachii* d-alanine-d-alanine ligase orthologue
1157348	5.074	28.54	UDP-glucose 4-epimerase
1186355	2.26	12.65	FAD-linked oxidase
1155959	6.55	34.31	MNNG and nitrosoguanidine resistance protein
1141103	32.82	162.69	trkA-N domain dehydrogenase
1107461	1.26	6.04	Chitinase
1187643	9.17	43.25	No annotation
1099871	4.00	18.48	Flavin-containing monooxygenase
1124492	3.68	16.34	Phosphoglycerate mutase
1135815	9.66	41.71	Serine/threonine kinase
52063	18.67	80.01	No annotation
1150465	11.56	48.79	No annotation
1161325	56.49	217.67	Integral membrane protein
1141963	25.56	92.48	Glutathione S-transferase
1145979	10.39	35.46	GMC oxidoreductase
Up-regulated genes in Δfum21			
1184413	92.14	22.85	Serine/threonine kinase
1184369	139.39	31.11	Lipase
1156756	32.65	6.04	No annotation
1185088	26.99	4.98	No annotation
1180662	357.01	65.37	Lipase
1181154	89.30	13.98	No annotation
1183897	535.00	81.16	Antifungal protein
1117716	35.428	5.03	Glycoside hydrolase family 7 protein CbhB
1187764	19.37	2.5476	No annotation
1146836	6.24	0.69	Hypothetical FAD/FMN-containing dehydrogenase
1164071	108.91	11.66	Peptidase G1, eqolisin

Both strains had been grown in TM for 16 h followed by 4 h in MM-X. Gene expression is expressed as Fragments Per Kilobase Of Exon Per Million Fragments Mapped (FPKM)

**Table 2:**
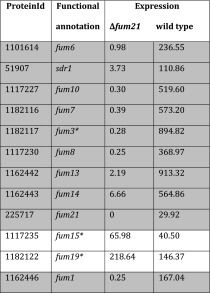
Expression of the genes of the fumonisin cluster in liquid shaken cultures of Δ*fum21* and the wild-type strain

Both strains had been grown in TM for 16 h followed by 4 h in MM-X. Gene expression is expressed as Fragments Per Kilobase Of Exon Per Million Fragments Mapped (FPKM). Gray shading indicates genes that are down-regulated in Δ*fum21* when compared to the wild-type strain. Asterisk indicates genes that are not significantly differentially expressed

Expression of the 74 differentially expressed genes in Δ*fum21* was assessed in the central, intermediate, and outer zones of wild-type and Δ*flbA* colonies using data of Krijgsheld and Wösten (2013). Six out of 11 up-regulated genes in Δ*fum21* were down-regulated in Δ*flbA* (Supplemental Table 3). Conversely, 10 out of the 63 down-regulated genes in Δ*fum21* were up-regulated in Δ*flbA*. In addition, 13 of the down-regulated genes in Δ*fum21* were also down-regulated in Δ*flbA* among which all genes of the fumonisin cluster (Supplemental Table 4). The genes in the fumonisin cluster were highly expressed at the periphery of xylose-grown colonies of *A. niger,* 6 of which were statistically significantly more highly expressed (i.e. exhibited > 2-fold higher expression levels) in this zone when compared to the more inner zones. Gene *fum21* was not differentially expressed in zones of the wild-type colonies.

## Discussion

Most wild-type *A. niger* strains produce fumonisin in liquid and solid cultures (Frisvad et al. 2007, 2011). Fumonisins are potent mycotoxins that exhibit neurotoxicity, hepatotoxicity, and nephrotoxicity in various animal models. They have also been linked to tumor formation in humans (esophageal cancer) and animals (Stockmann-Juvala and Savolainen 2008). The fumonisin biosynthesis cluster of *A. niger* has been proposed to originate from a horizontal gene transfer event (Khaldi and Wolfe 2011). The *Fusarium* cluster consists of up to 17 genes depending on the species (Proctor et al. 2003, 2008; Brown et al. 2007; Wiemann et al. 2013). Of these genes, expression of at least *fum1* and *fum8* is controlled by the transcriptional regulator Fum21 (Brown et al. 2007). A bidirectional BLAST showed that gene An01g06900 of *A. niger* is the orthologue of *fum21* of *Fusarium*. Inactivation of *fum21* in *A. niger* did not impact vegetative growth, mycelium morphology, conidiation, and (spatial) secretion of proteins. In contrast, Δ*fum21* of *A. niger* did not produce fumonisin, while expression of 10 out of 12 genes of the fumonisin gene cluster was reduced. These data show that Fum21 controls fumonisin production as was previously shown in *Fusarium* (Brown et al. 2007).

The function of Fum21 of *A. niger* and *Fusarium verticillioides* is remarkably similar despite the evolutionary distance between these species that belong to the Sordariomycetes and the Eurotiomycetes, respectively. Both proteins activate fumonisin production, while not affecting other processes such as growth and sporulation (Brown et al. 2007). In addition, both *fum21* homologs are regulated by genes involved in asexual development; i.e. the VeA homolog FvVE1 in *F. verticillioides* (Myung et al. 2009) and *flbA* in *A. niger.* It should be noted that deletion of *fum21* in *A. niger* completely abolished fumonisin production, while some fumonisin can still be detected in the *fum21* deletion strain of *F. verticillioides*. In the latter case the transcription factor genes *pac1* and *zfr1* also impact biosynthesis of this secondary metabolite (Shim and Woloshuk 2001; Flaherty et al. 2003; Flaherty and Woloshuk 2004; Bluhm and Woloshuk 2006). *A. niger* has orthologues of both genes. Future studies should confirm a role of these genes in fumonisin production. They might for instance regulate *fum15* and *fum19* that were shown not to be regulated by *fum21.* Results also showed that Fum21 of *A. niger* impacts production of the secondary metabolite pyranonigrin A. Indeed, absence of Fum21 of *A. niger* affected expression of genes of three other secondary metabolite clusters. Whether Fum21 of *F. verticillioides* also affects expression of other secondary metabolite clusters is not known.

As mentioned above, *fum21* is down-regulated in ∆*flbA* (Krijgsheld and Wösten 2013). This implies that FlbA is not only involved in conidiation, spatial secretion of proteins, composition of the secretome, cell wall architecture, and lysis of hyphae (Krijgsheld et al. 2013b) but also in controlling secondary metabolism. Indeed, all genes of the fumonisin cluster are down-regulated in ∆*flbA* (Krijgsheld and Wösten 2013). The fact that Fum21 does not control expression of 2 out of 12 genes of the cluster implies that FlbA impacts expression of another transcription factor involved in fumonisin production. Of interest, the *pac1* homologue of *A. niger* (known as pacC) is up-regulated in ∆*flbA* (Krijgsheld and Wösten 2013). Possibly, this transcription factor is a repressor of genes in this mycotoxin cluster.

LaeA was initially identified as a regulator of secondary metabolism in *A. nidulans* (Bok and Keller 2004). It controls expression of several gene clusters, including clusters involved in production of sterigmatocystin, penicillin, and lovastatin. LaeA is also involved in secondary metabolism in *A. niger*. It does not impact fumonisin production but it represses production of the compounds BMS-192548 and aspernigrin A, while activating production of asperrubrol, atromentin, and JBIR86 (Niu et al. 2015). Experimental data showed that production of aurasperone B, funalenone, kotanin, and tensidol are neither regulated by Fum21 (this work) nor by LaeA (Niu et al. 2015). This implies that other regulatory genes are involved in the production of these secondary metabolites. The fact that expression of *laeA* is not affected by FlbA in *A. niger* implies that its regulation is different from that of *fum21.* Together, these results indicate that *fum21, laeA,* and other transcriptional regulators are involved in secondary metabolite production in *A. niger*. These genes are potential targets to improve *A. niger* as a cell factory by minimizing production of mycotoxins.

## Electronic supplementary material

Below is the link to the electronic supplementary material.

Supplementary material 1 (DOCX 545 kb)
