# Supplementary material for: The FlbA-regulated predicted transcription factor Fum21 of Aspergillus niger is involved in fumonisin production
Source: Antonie Van Leeuwenhoek. 2017 Sep 30;111(3):311–22. doi: 10.1007/s10482-017-0952-1 (PMC5816093; doi:10.1007/s10482-017-0952-1)
Supplement: Supplementary file 1 — Supplementary material 1 (DOCX 545 kb) [file 10482_2017_952_MOESM1_ESM.docx]

**Supplemental Table 1.** Primers used in this study

| **Primer** |  |
| --- | --- |
| 1 | AGTATTCCACTCAGCTCTGGACTAACCTCTTTAATGGGATACTACCAGCCAGTAGTACC |
| 2 | TGATGTGTGTGACGTGATGTGTATATCCTACACTTAGTAAGACGCACGTTCCCC |
| 3 | TGATGATGGATATATGGAAGCTGGAGGATGCCCGTTTCCTATTAAGCAGTGCAGTG |
| 4 | TAGCAGGTGAGAACTCACTTGTACTCGACTCCTGCAGATACAGTATTGGACGGTGGCTGG |
| 5 | AAGCCGCTGCTGGAATTGGGCTCTGAGGTGCAGTGGAT |
| 6 | CGATGGATAATTGTGCCGTGTTGGGTGTTACGGAGCATTCA |
| 7 | GGCGTCGGTTTCCACTATC |
| 8 | AAAGTTCGACAGCGTCTCC |
| 9 | TCTCTAGATTTCGGCACGGCTAC |
| 10 | TGACACAGTACACGAGGACTTC |
| 11 | TGATTACGAATTCCCTCGGGGAGTCATATGAAAGG |
| 12 | GACGCGTGGATCCCCGCGGGTAATATTGAGCCAAC |
| 13 | TTCTGGCATGCGGAGAGAC |
| 14 | GTGGTACCATGGGTTGAGTGG |
| 15 | TACGACGATAAGCCATCCGC |
| 16 | ACAACTGTCGAAGGGGCATT |
| 17 | GACTGAGAGTGTACGCCTGG |
| 18 | GCCTGTATAGACGCCATGCT |
| 19 | TAGCGATTGTTGGCATGGGT |
| 20 | GTCCACGTTGAACCGACTCT |
| 21 | ATGATGGCTGCCTCTGACTTC |
| 22 | TTCTTGCTCTGGATGTTGCG |
| 23 | GCTCGGCACCTTACGAGAAATC |
| 24 | TTCAGCCTTGCGACCATACTCC |

**Supplemental Table 2.** Differentially expressed genes in Δ*fum21* compared to the wild-type strain in MM-X (Δ21XX and wtXX) or MM-G (Δ21GX and wtGX). Expression levels in FPKM are the average of duplicates. Rows of genes implied in secondary metabolism are grey-shaded.

| **ProteinId** | **Δ21XX** | **Δ21GX** | **wtXX** | **wtGX** | **Functional annotation** | **PFAM annotation** | **GO annotation** | **KEGG annotation** | **Predicted secretion signal sequence** | **Secondary metabolism** |
| --- | --- | --- | --- | --- | --- | --- | --- | --- | --- | --- |
| **DOWN REGULATED IN Δ*fum21*** | | | | | | | | | | |
| 1166045 | 0 | 0 | 5.49 | 13.21 | AAA+-type ATPase |  | GO:0005524\| ATP binding |  |  |  |
| 1182124 | 0 | 0 | 2.90 | 4.89 | Major Facilitator Superfamily transporter | PF07690,11\|MFS_1;PF07690,11\|MFS_1;PF01306,14\|LacY_symp | GO:0016020\|membrane;GO:0006810\|transport;GO:0016021\|integral component of membrane;GO:0055085\|transmembrane transport |  |  | Part of secondary metabolism cluster 15;Secondary metabolism, Type: Decorating |
| 225717 | 0.02 | 0 | 25.19 | 29.92 | Transcription factor An01g06900 | PF04082,13\|Fungal_trans;PF00172,13\|Zn_clus | GO:0000981\|sequence-specific DNA binding RNA polymerase II transcription factor activity;GO:0006355\|regulation of transcription, DNA-templated;GO:0003677\|DNA binding;GO:0005634\|nucleus;GO:0008270\|zinc ion binding;GO:0006351\|transcription, DNA-templated |  |  | Part of secondary metabolism cluster 15;Secondary metabolism, Type: Decorating |
| 1117227 | 0.06 | 0.30 | 285.71 | 519.60 | Peroxisomal acyl-CoA synthetase; fum10 orthologue | PF00501,23\|AMP-binding;PF13193,1\|AMP-binding_C | GO:0003824\|catalytic activity;GO:0008152\|metabolic process |  |  | Part of secondary metabolism cluster 15 |
| 1117230 | 0.12 | 0.25 | 224.46 | 368.97 | α-oxoamine synthase; serine palmitoyltransferase; fum8 orthologue | PF00155,16\|Aminotran_1_2 | GO:0009058\|biosynthetic process;GO:0030170\|pyridoxal phosphate binding | Metabolism\|Lipid metabolism\|Sphingolipid metabolism\|K00654\|E2,3,1,50, serine palmitoyltransferase [EC:2,3,1,50]:EC:2,3,1,50 |  | Part of secondary metabolism cluster 15;Secondary metabolism, Type: Decorating |
| 1182116 | 0.12 | 0.39 | 347.32 | 573.20 | Fe-containing alcohol dehydrogenase type IV; fum7 orthologue | PF00465,14\|Fe-ADH | GO:0016491\|oxidoreductase activity;GO:0046872\|metal ion binding;GO:0055114\|oxidation-reduction process | Metabolism\|Carbohydrate metabolism\|Glycolysis / Gluconeogenesis\|K04022\|eutG, alcohol dehydrogenase: |  | Part of secondary metabolism cluster 15 |
| 1142053 | 1.179 | 1.0923 | 2068.63 | 1433.24 | No annotation |  |  |  |  |  |
| 1162446 | 0.14 | 0.25 | 81.29 | 167.04 | Polyketide synthase; fum1 orthologue | PF00698,16\|Acyl_transf_1;PF08659,5\|KR;PF14765,1\|PS-DH;PF00109,21\|ketoacyl-synt;PF00106,20\|adh_short;PF02801,17\|Ketoacyl-synt_C;PF08242,7\|Methyltransf_12;PF00107,21\|ADH_zinc_N;PF13489,1\|Methyltransf_23;PF13602,1\|ADH_zinc_N_2;PF13847,1\|Methyltransf_31;PF12847,2\|Methyltransf_18;PF08240,7\|ADH_N;PF00550,20\|PP-binding;PF08241,7\|Methyltransf_11 | GO:0016491\|oxidoreductase activity;GO:0008270\|zinc ion binding;GO:0055114\|oxidation-reduction process;GO:0008168\|methyltransferase activity;GO:0008152\|metabolic process | Metabolism\|Overview\|Fatty acid metabolism\|K00665\|FASN, fatty acid synthase, animal type [EC:2,3,1,85]:EC:2,3,1,85;Metabolism\|Lipid metabolism\|Fatty acid biosynthesis\|K00665\|FASN, fatty acid synthase, animal type [EC:2,3,1,85]:EC:2,3,1,85;Environmental Information Processing\|Signal transduction\|AMPK signaling pathway\|K00665\|FASN, fatty acid synthase, animal type [EC:2,3,1,85]:EC:2,3,1,85;Organismal Systems\|Endocrine system\|Insulin signaling pathway\|K00665\|FASN, fatty acid synthase, animal type [EC:2,3,1,85]:EC:2,3,1,85 |  | Part of secondary metabolism cluster 15;Secondary metabolism, Type: PKS |
| 1162442 | 1.66 | 2.19 | 807.36 | 913.32 | NAD-dependent epimerase/dehydratase; fum13 orthologue | PF01370,16\|Epimerase;PF13460,1\|NAD_binding_10;PF01073,14\|3Beta_HSD;PF02719,10\|Polysacc_synt_2;PF00106,20\|adh_short;PF08659,5\|KR;PF07993,7\|NAD_binding_4;PF05368,8\|NmrA | GO:0003854\|3-beta-hydroxy-delta5-steroid dehydrogenase activity;GO:0016491\|oxidoreductase activity;GO:0055114\|oxidation-reduction process;GO:0044237\|cellular metabolic process;GO:0008152\|metabolic process;GO:0050662\|coenzyme binding;GO:0006694\|steroid biosynthetic process;GO:0009058\|biosynthetic process;GO:0003824\|catalytic activity;GO:0016616\|oxidoreductase activity, acting on the CH-OH group of donors, NAD or NADP as acceptor |  |  | Part of secondary metabolism cluster 15;Secondary metabolism, Type: Decorating |
| 1101614 | 0.45 | 0.98 | 145.98 | 236.55 | Cytochrome p450; fum6 orthologue | PF00067,17\|p450;PF00667,15\|FAD_binding_1;PF00258,20\|Flavodoxin_1;PF00175,16\|NAD_binding_1 | GO:0016705\|oxidoreductase activity, acting on paired donors, with incorporation or reduction of molecular oxygen;GO:0020037\|heme binding;GO:0016491\|oxidoreductase activity;GO:0055114\|oxidation-reduction process;GO:0010181\|FMN binding;GO:0005506\|iron ion binding | Metabolism\|Lipid metabolism\|Fatty acid degradation\|K14338\|cypD_E, CYP102A2_3, cytochrome P450 / NADPH-cytochrome P450 reductase [EC:1,14,14,1 1,6,2,4]:EC:1,14,14,1 1,6,2,4;Metabolism\|Amino acid metabolism\|Tryptophan metabolism\|K14338\|cypD_E, CYP102A2_3, cytochrome P450 / NADPH-cytochrome P450 reductase [EC:1,14,14,1 1,6,2,4]:EC:1,14,14,1 1,6,2,4;Metabolism\|Xenobiotics biodegradation and metabolism\|Aminobenzoate degradation\|K14338\|cypD_E, CYP102A2_3, cytochrome P450 / NADPH-cytochrome P450 reductase [EC:1,14,14,1 1,6,2,4]:EC:1,14,14,1 1,6,2,4 |  | Part of secondary metabolism cluster 15;Secondary metabolism, Type: Decorating |
| 1142051 | 4.63 | 8.34 | 1192.75 | 1607.79 | No annotation |  |  |  |  |  |
| 1166044 | 2.66 | 2.75 | 284.01 | 347.93 | No annotation |  |  |  |  |  |
| 1172265 | 1.70 | 1.62 | 3.51 | 191.62 | Oxidoreductase | PF03171,15\|2OG-FeII_Oxy;PF14226,1\|DIOX_N | GO:0016706\|oxidoreductase activity, acting on paired donors, with incorporation or reduction of molecular oxygen, 2-oxoglutarate as one donor, and incorporation of one atom each of oxygen into both donors;GO:0055114\|oxidation-reduction process;GO:0016491\|oxidoreductase activity |  |  |  |
| 1162443 | 3.61 | 6.66 | 384.23 | 564.86 | CoA-dependent acyltransferase; fum14 orthologue | PF00668,15\|Condensation;PF00550,20\|PP-binding |  |  |  | Part of secondary metabolism cluster 15 |
| 1186369 | 8.13 | 0.57 | 11.86 | 19.06 | Ca2+-modulated nonselective cation channel polycystin; *Homo sapiens* mucin 5AC orthologue |  |  |  |  |  |
| 51907 | 1.45 | 3.73 | 75.16 | 110.86 | Predicted 3-ketosphinganine reductase | PF00106,20\|adh_short | GO:0016491\|oxidoreductase activity;GO:0008152\|metabolic process |  |  | Part of secondary metabolism cluster 15;Secondary metabolism, Type: Decorating |
| 1181633 | 11.77 | 0.49 | 73.92 | 12.69 | Putative SWI-SNF chromatin-remodeling complex protein |  |  |  |  | Part of secondary metabolism cluster 12 |
| 1082505 | 1.56 | 1.48 | 18.44 | 32.99 | Major Facilitator Superfamily permease | PF07690,11\|MFS_1;PF00083,19\|Sugar_tr | GO:0016021\|integral component of membrane;GO:0055085\|transmembrane transport; GO:0022857\| transmembrane transporter activity |  |  |  |
| 1142861 | 2.77 | 1.59 | 34.50 | 33.92 | Chloroperoxidase | PF01328,12\|Peroxidase_2 | GO:0004601\|peroxidase activity |  |  |  |
| 1159889 | 0.88 | 0.93 | 20.02 | 17.88 | O-methyltransferase | PF00891,13\|Methyltransf_2 | GO:0008171\|O-methyltransferase activity | Metabolism\|iosynthesis of other secondary metabolites\|Phenylpropanoid biosynthesis\|K13066\|E2,1,1,68, COMT, caffeic acid 3-O-methyltransferase [EC:2,1,1,68]:EC:2,1,1,68 |  | Part of secondary metabolism cluster 62;Secondary metabolism, Type: Decorating |
| 1087288 | 1.98 | 2.37 | 82.10 | 39.12 | Taurine catabolism dioxygenase TauD | PF02668,11\|TauD | GO:0016491\|oxidoreductase activity; GO:0055114\|oxidation-reduction process |  |  |  |
| 45784 | 12.44 | 1.70 | 21.20 | 25.17 | Ca^2+^-modulated nonselective cation channel polycystin |  |  |  | yes |  |
| 1169210 | 2.53 | 5.86 | 12.61 | 72.09 | Glutathione S-transferase-like protein | PF14497,1\|GST_C_3;PF13410,1\|GST_C_2 |  |  |  |  |
| 1116476 | 1.65 | 2.31 | 3.80 | 28.18 | CDR ABC transporter | PF01061,19\|ABC2_membrane;PF01061,19\|ABC2_membrane;PF00005,22\|ABC_tran;PF00005,22\|ABC_tran;PF06422,7\|PDR_CDR;PF12698,2\|ABC2_membrane_3 | GO:0016887\|ATPase activity; GO:0016021\|integral component of membrane;GO:0016020\|membrane;GO:0005524\|ATP binding; GO:0042626\| ATPase activity, coupled to transmembrane movement of substances;GO:0006810\|transport | Environmental Information Processing\|Membrane transport\|ABC transporters\|K08712\|ABCG2,SNQ2, ATP-binding cassette, subfamily G (WHITE), member 2, SNQ2: |  | Part of secondary metabolism cluster 12;Secondary metabolism, Type: Decorating |
| 1089440 | 12.82 | 2.52 | 48.93 | 29.01 | Major Facillitator Superfamily transporter | PF07690,11\|MFS_1 | GO:0016021\|integral component of membrane;GO:0055085\| transmembrane transport |  |  |  |
| 1112167 | 0.46 | 0.60 | 1.10 | 6.38 | Polyketide synthase AdaA | PF00109,21\|ketoacyl-synt;PF00698,16\|Acyl_transf_1;PF02801,17\|Ketoacyl-synt_C;PF00550,20\|PP-binding;PF00108,18\|Thiolase_N | GO:0008152\|metabolic process; GO:0016747\|transferase activity, transferring acyl groups other than amino-acyl groups |  |  | Part of secondary metabolism cluster 70;Secondary metabolism, Type: PKS |
| 1181632 | 7.20 | 2.79 | 88.83 | 26.80 | No annotation |  |  |  |  | Part of secondary metabolism cluster 12 |
| 1115620 | 2.08 | 1.27 | 2.78 | 12.02 | C-type lectin |  |  |  | yes |  |
| 189113 | 21.33 | 8.60 | 4.66 | 80.47 | NmrA-like family protein | PF13460,1\|NAD_binding_10;PF05368,8\|NmrA |  |  |  | Part of secondary metabolism cluster 1 |
| 1005100 | 31.19 | 2.33 | 202.62 | 21.65 | No annotation |  |  |  |  | Part of secondary metabolism cluster 12 |
| 1186279 | 23.46 | 20.64 | 77.62 | 191.85 | No annotation | PF09351,5\|DUF1993 |  |  |  |  |
| 1103854 | 8.96 | 1.34 | 16.66 | 11.77 | Glycosyl transferase | PF00534,15\|Glycos_transf_1;PF11997,3\|DUF3492 | GO:0009058\|biosynthetic process |  |  |  |
| 1139199 | 3.10 | 4.02 | 7.23 | 34.97 | Mono-oxygenase, FAD-binding/aromatic ring hydroxylase | PF01494,14\|FAD_binding_3 |  |  |  | Part of secondary metabolism cluster 70;Secondary metabolism, Type: Decorating |
| 1186352 | 11.54 | 2.74 | 3.97 | 23.65 | Molecular chaperone | PF00011,16\|HSP20 |  | Genetic Information Processing\|Folding, sorting and degradation\|Protein processing in endoplasmic reticulum\|K13993\|HSP20, HSP20 family protein: |  |  |
| 1109756 | 1.87 | 1.59 | 8.34 | 13.54 | No annotation | PF11001,3\|DUF2841 |  |  |  |  |
| 1187549 | 2.13 | 1.99 | 41.60 | 16.75 | Integral membrane protein |  |  |  |  |  |
| 1125454 | 210.50 | 0.71 | 6.22 | 5.80 | Dihydroxy-acid dehydratase | PF00920,16\|ILVD_EDD | GO:0003824\|catalytic activity;GO:0008152\|metabolic process | Metabolism\|Overview\|2-Oxocarboxylic acid metabolism\|K01687\|ilvD, dihydroxy-acid dehydratase [EC:4,2,1,9]:EC:4,2,1,9;Metabolism\|Overview\|Biosynthesis of amino acids\|K01687\|ilvD, dihydroxy-acid dehydratase [EC:4,2,1,9]:EC:4,2,1,9;Metabolism\|Amino acid metabolism\|Valine, leucine and isoleucine biosynthesis\|K01687\|ilvD, dihydroxy-acid dehydratase [EC:4,2,1,9]:EC:4,2,1,9;Metabolism\|Metabolism of cofactors and vitamins\|Pantothenate and CoA biosynthesis\|K01687\|ilvD, dihydroxy-acid dehydratase [EC:4,2,1,9]:EC:4,2,1,9 |  |  |
| 1184525 | 2.96 | 3.92 | 16.19 | 31.30 | Non-ribosomal peptide synthetase | PF00501,23\|AMP-binding;PF07993,7\|NAD_binding_4;PF01370,16\|Epimerase;PF00550,20\|PP-binding | GO:0003824\|catalytic activity;GO:0050662\|coenzyme binding;GO:0044237\|cellular metabolic process;GO:0008152\|metabolic process | Metabolism\|Overview\|Biosynthesis of amino acids\|K00143\|LYS2, L-aminoadipate-semialdehyde dehydrogenase [EC:1,2,1,31]:EC:1,2,1,31;Metabolism\|Amino acid metabolism\|Lysine biosynthesis\|K00143\|LYS2, L-aminoadipate-semialdehyde dehydrogenase [EC:1,2,1,31]:EC:1,2,1,31;Metabolism\|Amino acid metabolism\|Lysine degradation\|K00143\|LYS2, L-aminoadipate-semialdehyde dehydrogenase [EC:1,2,1,31]:EC:1,2,1,31 |  | Part of secondary metabolism cluster 35;Secondary metabolism, Type: NRPS-like |
| 1124090 | 4.10 | 2.40 | 14.39 | 18.02 | Tryptophan synthase | PF00290,15\|Trp_syntA;PF00291,20\|PALP | GO:0004834\|tryptophan synthase activity;GO:0006568\|tryptophan metabolic process | Metabolism\|Overview\|Biosynthesis of amino acids\|K01694\|TRP, tryptophan synthase [EC:4,2,1,20]:EC:4,2,1,20;Metabolism\|Amino acid metabolism\|Glycine, serine and threonine metabolism\|K01694\|TRP, tryptophan synthase [EC:4,2,1,20]:EC:4,2,1,20;Metabolism\|Amino acid metabolism\|Phenylalanine, tyrosine and tryptophan biosynthesis\|K01694\|TRP, tryptophan synthase [EC:4,2,1,20]:EC:4,2,1,20 |  |  |
| 1015414 | 2.08 | 1.95 | 8.27 | 14.50 | Short chain dehygrogenase | PF00106,20\|adh_short;PF13561,1\|adh_short_C2;PF08659,5\|KR | GO:0016491\|oxidoreductase activity;GO:0008152\|metabolic process | Metabolism\|Overview\|Fatty acid metabolism\|K00059\|fabG, 3-oxoacyl-[acyl-carrier protein] reductase [EC:1,1,1,100]:acyl-carrier protein;Metabolism\|Lipid metabolism\|Fatty acid biosynthesis\|K00059\|fabG, 3-oxoacyl-[acyl-carrier protein] reductase [EC:1,1,1,100]:acyl-carrier protein;Metabolism\|Lipid metabolism\|Biosynthesis of unsaturated fatty acids\|K00059\|fabG, 3-oxoacyl-[acyl-carrier protein] reductase [EC:1,1,1,100]:acyl-carrier protein;Metabolism\|Metabolism of cofactors and vitamins\|Biotin metabolism\|K00059\|fabG, 3-oxoacyl-[acyl-carrier protein] reductase [EC:1,1,1,100]:acyl-carrier protein |  |  |
| 1139200 | 21.10 | 33.67 | 39.78 | 239.67 | AdaD | PF13847,1\|Methyltransf_31;PF13489,1\|Methyltransf_23;PF08241,7\|Methyltransf_11 | GO:0008152\|metabolic process; GO:0008168\| methyltransferase activity |  |  | Part of secondary metabolism cluster 70 |
| 1187587 | 1.67 | 2.19 | 6.99 | 15.55 | AAA+-type ATPase | PF00004,24\|AAA | GO:0005524\|ATP binding |  |  |  |
| 1152279 | 0.36 | 4.10 | 0.23 | 28.71 | Major Facilitator Superfamily transporter | PF07690,11\|MFS_1;PF00083,19\|Sugar_tr | GO:0016021\|integral component of membrane;GO:0055085\|transmembrane transport; GO:0022857\| transmembrane transporter activity |  |  |  |
| 1162650 | 11.45 | 21.21 | 5.68 | 146.94 | Aegerolysin | PF06355,8\|Aegerolysin | GO:0019836\|hemolysis by symbiont of host erythrocytes |  |  |  |
| 1200239 | 3.73 | 5.01 | 4.02 | 34.01 | NmrA-like family protein | PF05368,8\|NmrA;PF13460,1\|NAD_binding_10 |  |  |  | Part of secondary metabolism cluster 1 |
| 1223842 | 2.06 | 1.36 | 9.52 | 9.10 | hypothetical FAD/FMN-containing dehydrogenase | PF01565,18\|FAD_binding_4;PF08031,7\|BBE | GO:0055114\|oxidation-reduction process;GO:0016491\|oxidoreductase activity;GO:0050660\|flavin adenine dinucleotide binding;GO:0008762\|UDP-N-acetylmuramate dehydrogenase activity |  | yes | Part of secondary metabolism cluster 64;Secondary metabolism, Type: Decorating |
| 1186845 | 1.03 | 0.79 | 1.10 | 5.13 | No annotation |  |  |  |  |  |
| 1187028 | 162.80 | 192.14 | 393.75 | 1228.87 | No annotation | PF08592,6\|DUF1772 |  |  |  |  |
| 1181350 | 2.29 | 3.78 | 5.74 | 21.89 | Aspergillus kawachii D-alanine-D-alanine ligase orthologue | PF13535,1\|ATP-grasp_4;PF07478,8\|Dala_Dala_lig_C | GO:0008716\|D-alanine-D-alanine ligase activity |  |  |  |
| 1157348 | 41.45 | 5.07 | 52.27 | 28.54 | UDP-glucose 4-epimerase | PF01370,16\|Epimerase;PF13950,1\|Epimerase_Csub;PF07993,7\|NAD_binding_4;PF01073,14\|3Beta_HSD;PF00106,20\|adh_short | GO:0003854\|3-beta-hydroxy-delta5-steroid dehydrogenase activity;GO:0016491\|oxidoreductase activity;GO:0055114\|oxidation-reduction process;GO:0044237\|cellular metabolic process;GO:0008152\|metabolic process;GO:0050662\|coenzyme binding;GO:0006694\|steroid biosynthetic process;GO:0003824\|catalytic activity;GO:0016616\|oxidoreductase activity, acting on the CH-OH group of donors, NAD or NADP as acceptor | Metabolism\|Carbohydrate metabolism\|Galactose metabolism\|K01784\|galE, GALE, UDP-glucose 4-epimerase [EC:5,1,3,2]:EC:5,1,3,2;Metabolism\|Carbohydrate metabolism\|Amino sugar and nucleotide sugar metabolism\|K01784\|galE, GALE, UDP-glucose 4-epimerase [EC:5,1,3,2]:EC:5,1,3,2 |  |  |
| 1186355 | 2.29 | 2.26 | 3.36 | 12.65 | FAD-linked oxidase | PF01565,18\|FAD_binding_4;PF08031,7\|BBE | GO:0055114\|oxidation-reduction process;GO:0016491\|oxidoreductase activity;GO:0050660\|flavin adenine dinucleotide binding;GO:0008762\|UDP-N-acetylmuramate dehydrogenase activity |  | yes |  |
| 1155959 | 15.68 | 6.55 | 23.19 | 34.31 | MNNG and nitrosoguanidine resistance protein | PF12051,3\|DUF3533 |  |  |  |  |
| 1141103 | 432.60 | 32.82 | 371.26 | 162.69 | trkA-N domain dehydrogenase | PF13460,1\|NAD_binding_10;PF01370,16\|Epimerase;PF05368,8\|NmrA | GO:0003824\|catalytic activity;GO:0050662\|coenzyme binding;GO:0044237\|cellular metabolic process |  |  | Part of secondary metabolism cluster 1 |
| 1107461 | 2.15 | 1.26 | 9.55 | 6.04 | Chitinase | PF12708,2\|Pectate_lyase_3;PF12708,2\|Pectate_lyase_3;PF01476,15\|LysM;PF01476,15\|LysM |  |  |  |  |
| 1187643 | 16.44 | 9.17 | 47.07 | 43.25 | No annotation | PF13894,1\|zf-C2H2_4 |  |  |  |  |
| 1099871 | 7.50 | 4.00 | 18.39 | 18.48 | Flavin-containing monooxygenase | PF13738,1\|Pyr_redox_3;PF00743,14\|FMO-like;PF13450,1\|NAD_binding_8;PF13434,1\|K_oxygenase | GO:0004499\|N,N-dimethylaniline monooxygenase activity;GO:0055114\|oxidation-reduction process;GO:0050661\|NADP binding;GO:0050660\|flavin adenine dinucleotide binding | Metabolism\|Amino acid metabolism\|Histidine metabolism\|K00492\|E1,14,13,-,:;Metabolism\|Metabolism of terpenoids and polyketides\|Limonene and pinene degradation\|K00492\|E1,14,13,-,:;Metabolism\|Xenobiotics biodegradation and metabolism\|Aminobenzoate degradation\|K00492\|E1,14,13,-,:;Metabolism\|Xenobiotics biodegradation and metabolism\|Chlorocyclohexane and chlorobenzene degradation\|K00492\|E1,14,13,-,:;Metabolism\|Xenobiotics biodegradation and metabolism\|Toluene degradation\|K00492\|E1,14,13,-,:;Metabolism\|Xenobiotics biodegradation and metabolism\|Bisphenol degradation\|K00492\|E1,14,13,-,:;Metabolism\|Xenobiotics biodegradation and metabolism\|Naphthalene degradation\|K00492\|E1,14,13,-,:;Metabolism\|Xenobiotics biodegradation and metabolism\|Polycyclic aromatic hydrocarbon degradation\|K00492\|E1,14,13,-,: |  |  |
| 1124492 | 3.56 | 3.68 | 16.86 | 16.34 | Phosphoglycerate mutase | PF00300,17\|His_Phos_1 |  | Metabolism\|Overview\|Carbon metabolism\|K15634\|gpmB, probable phosphoglycerate mutase [EC:5,4,2,12]:EC:5,4,2,12;Metabolism\|Overview\|Biosynthesis of amino acids\|K15634\|gpmB, probable phosphoglycerate mutase [EC:5,4,2,12]:EC:5,4,2,12;Metabolism\|Carbohydrate metabolism\|Glycolysis / Gluconeogenesis\|K15634\|gpmB, probable phosphoglycerate mutase [EC:5,4,2,12]:EC:5,4,2,12;Metabolism\|Energy metabolism\|Methane metabolism\|K15634\|gpmB, probable phosphoglycerate mutase [EC:5,4,2,12]:EC:5,4,2,12;Metabolism\|Amino acid metabolism\|Glycine, serine and threonine metabolism\|K15634\|gpmB, probable phosphoglycerate mutase [EC:5,4,2,12]:EC:5,4,2,12 |  |  |
| 1135815 | 44.48 | 9.66 | 78.96 | 41.71 | Serine/threonine kinase | PF00069,20\|Pkinase;PF07714,12\|Pkinase_Tyr | GO:0005524\|ATP binding;GO:0004672\|protein kinase activity;GO:0006468\|protein phosphorylation |  |  |  |
| 52063 | 17.45 | 18.67 | 99.88 | 80.01 | No annotation |  |  |  |  |  |
| 1150465 | 8.758 | 11.56 | 39.42 | 48.79 | No annotation | PF14420,1\|Clr5 |  |  |  |  |
| 1161325 | 36.78 | 56.49 | 164.01 | 217.67 | Integral membrane protein |  |  |  |  |  |
| 1141963 | 55.66 | 25.56 | 65.19 | 92.48 | Glutathione S-transferase | PF13417,1\|GST_N_3;PF00043,20\|GST_C;PF02798,15\|GST_N | GO:0005515\|protein binding | Metabolism\|Metabolism of other amino acids\|Glutathione metabolism\|K00799\|GST, gst, glutathione S-transferase [EC:2,5,1,18]:EC:2,5,1,18;Metabolism\|Xenobiotics biodegradation and metabolism\|Metabolism of xenobiotics by cytochrome P450\|K00799\|GST, gst, glutathione S-transferase [EC:2,5,1,18]:EC:2,5,1,18;Metabolism\|Xenobiotics biodegradation and metabolism\|Drug metabolism - cytochrome P450\|K00799\|GST, gst, glutathione S-transferase [EC:2,5,1,18]:EC:2,5,1,18;Human Diseases\|Cancers\|Chemical carcinogenesis\|K00799\|GST, gst, glutathione S-transferase [EC:2,5,1,18]:EC:2,5,1,18 |  |  |
| 1145979 | 23.89 | 10.39 | 47.82 | 35.46 | GMC oxidoreductase | PF00732,14\|GMC_oxred_N;PF05199,8\|GMC_oxred_C;PF01266,19\|DAO | GO:0016491\|oxidoreductase activity;GO:0016614\|oxidoreductase activity, acting on CH-OH group of donors;GO:0055114\|oxidation-reduction process;GO:0050660\|flavin adenine dinucleotide binding | Metabolism\|Amino acid metabolism\|Glycine, serine and threonine metabolism\|K00108\|E1,1,99,1, betA, CHDH, choline dehydrogenase [EC:1,1,99,1]:EC:1,1,99,1 |  | Part of secondary metabolism cluster 46 |
| **UPREGULATED IN Δ*fum21*** | | | | | | | | | | |
| 1184413 | 32.46 | 92.14 | 16.80 | 22.85 | Serine/threonine kinase |  |  |  |  |  |
| 1184369 | 12.91 | 139.39 | 15.53 | 31.11 | Lipase | PF00657,17\|Lipase_GDSL | GO:0006629\|lipid metabolic process;GO:0016788\|hydrolase activity, acting on ester bonds |  | yes |  |
| 1156756 | 73.37 | 32.65 | 18.31 | 6.04 | No annotation |  |  |  |  |  |
| 1185088 | 41.49 | 26.99 | 10.13 | 4.98 | No annotation |  |  |  | yes |  |
| 1180662 | 209.40 | 357.01 | 52.59 | 65.37 | Lipase | PF01764,20\|Lipase_3;PF03893,11\|Lipase3_N | GO:0006629\|lipid metabolic process;GO:0004806\|triglyceride lipase activity;GO:0016042\|lipid catabolic process | Metabolism\|iosynthesis of other secondary metabolites\|Tropane, piperidine and pyridine alkaloid biosynthesis\|K01066\|E3,1,1,-, esterase / lipase [EC:3,1,1,-]:EC:3,1,1,-;Metabolism\|Xenobiotics biodegradation and metabolism\|Bisphenol degradation\|K01066\|E3,1,1,-, esterase / lipase [EC:3,1,1,-]:EC:3,1,1,- | yes |  |
| 1181154 | 176.00 | 89.30 | 43.13 | 13.98 | No annotation |  |  |  |  |  |
| 1183897 | 11.41 | 535.00 | 17.59 | 81.16 | Antifungal protein | PF11402,3\|Antifungal_prot |  |  | yes |  |
| 1117716 | 4.09 | 35.43 | 2.23 | 5.03 | Glycoside Hydrolase Family 7 protein CbhB | PF00840,15\|Glyco_hydro_7;PF00734,13\|CBM_1 | GO:0030248\|cellulose binding;GO:0005576\|extracellular region;GO:0005975\|carbohydrate metabolic process;GO:0004553\|hydrolase activity, hydrolyzing O-glycosyl compounds |  | yes |  |
| 1187764 | 25.43 | 19.37 | 8.72 | 2.55 | No annotation |  |  |  |  |  |
| 1146836 | 3.82 | 6.24 | 2.71 | 0.69 | hypothetical FAD/FMN-containing dehydrogenase | PF01565,18\|FAD_binding_4;PF08031,7\|BBE | GO:0055114\|oxidation-reduction process;GO:0016491\|oxidoreductase activity;GO:0050660\|flavin adenine dinucleotide binding;GO:0008762\|UDP-N-acetylmuramate dehydrogenase activity |  | yes |  |
| 1164071 | 96.22 | 108.91 | 30.43 | 11.66 | Peptidase G1, eqolisin | PF01828,12\|Peptidase_A4 | GO:0006508\|proteolysis;GO:0004190\|aspartic-type endopeptidase activity |  | yes |  |

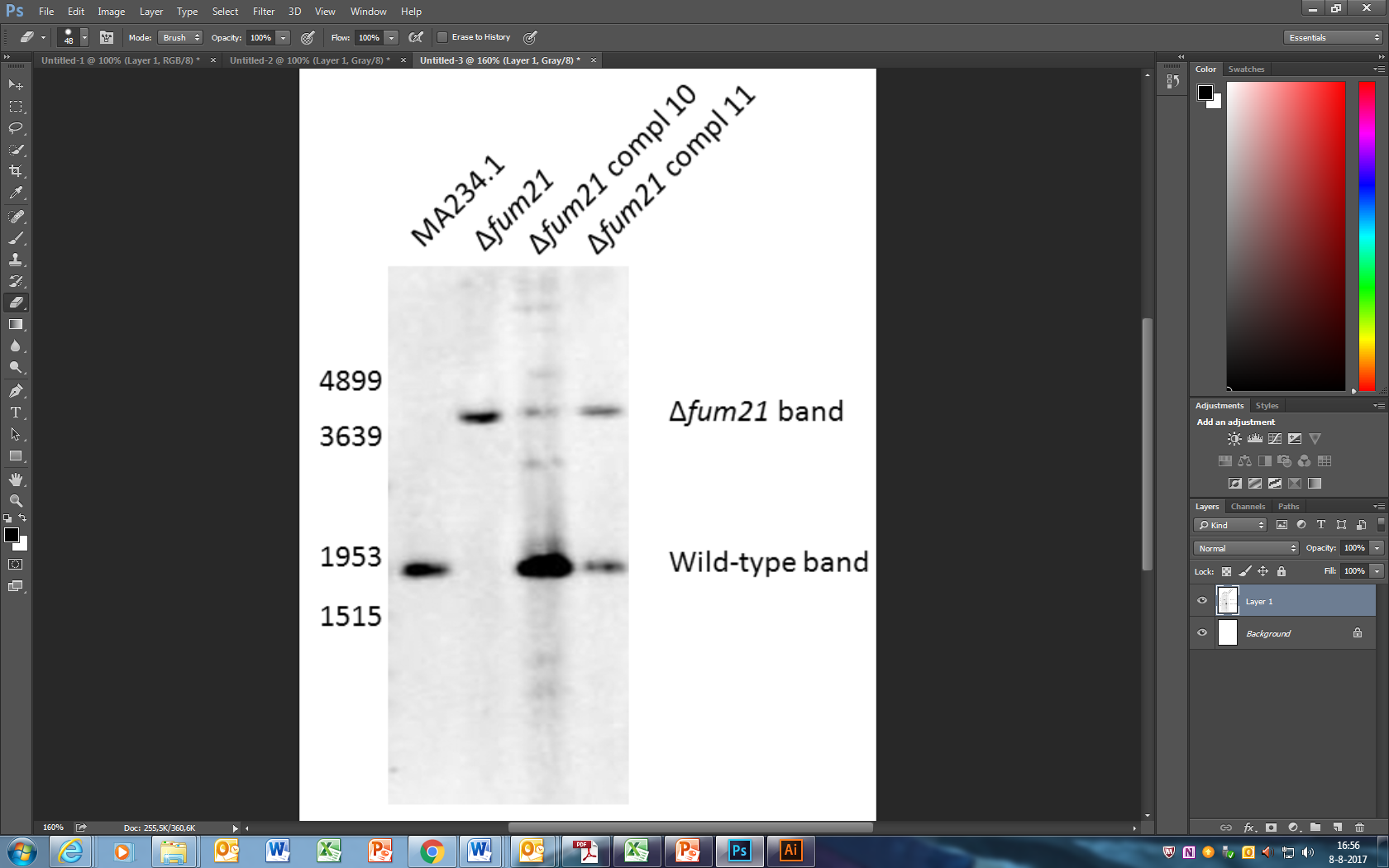


**Supplemental Figure 1.** Southern blot analysis of wild type strain MA234.1, deletion strain Δ*fum21* and complemented Δ*fum21* strains 10 and 11. Deletion of *fum21* eliminated a HindIII site, thereby giving a fragment of 4075 and 1876 base pairs in Δ*fum21* and wild type, respectively. Complemented strain 10 has multiple copies of *fum21,* while complemented strain 11 has a single integrated copy in its genome.

**
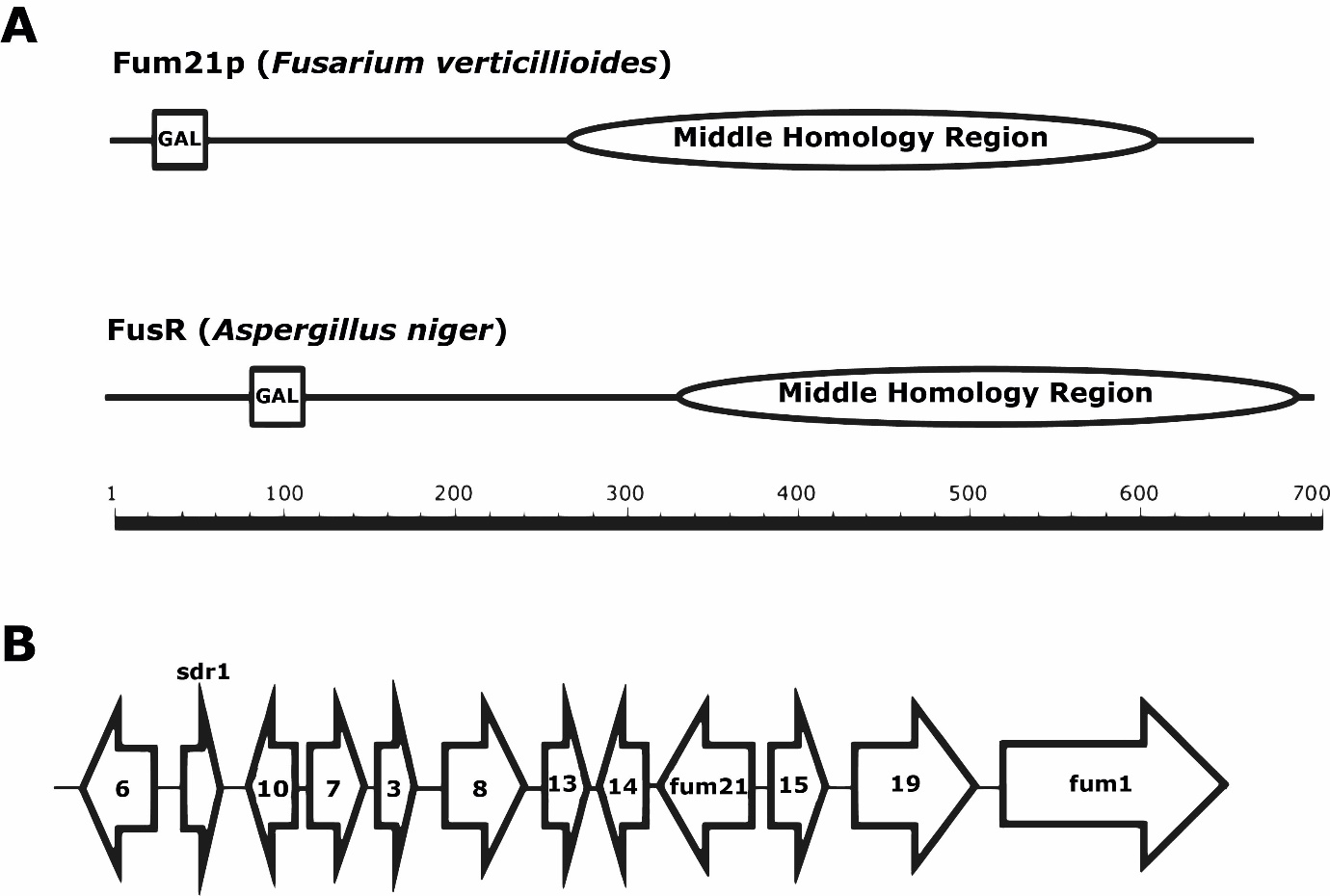
**

**Fum21 (*Aspergillus niger)***

**Supplemental Figure 2.** Schematic overview of Fum21 of *Fusarium vertillioides* and *A. niger* (A) and the fumonisin gene cluster of *A. niger* according to Susca et al (2014) (B). GAL = GAL4-like DNA binding domain.
